# Does Gender Matter in Non-Hodgkin Lymphoma? Differences in Epidemiology, Clinical Behavior, and Therapy

**DOI:** 10.5041/RMMJ.10172

**Published:** 2014-10-29

**Authors:** Nurit Horesh, Netanel A. Horowitz

**Affiliations:** 1 Department of Internal Medicine H, Rambam Health Care Campus, Haifa, Israel;; 2 Department of Hematology and Bone Marrow Transplantation, Rambam Health Care Campus, Haifa, Israel; 3 Bruce Rappaport Faculty of Medicine, Technion–Israel Institute of Technology, Haifa, Israel

**Keywords:** Gender, non-Hodgkin lymphoma, rituximab

## Abstract

Non-Hodgkin lymphoma (NHL) is one of the most common hematologic malignancies worldwide. The incidence of NHL has been rising for several decades; however, in the last 20 years, it reached a plateau. NHL incidence among males is significantly higher than in females. In addition to gender itself, gravidity has a protective role against NHL occurrence. Gender also matters in terms of NHL clinical characteristics. For example, female predominance was found in three extra-nodal sites (the breast, thyroid, and the respiratory system) occasionally involved in NHL. The diagnosis of NHL during pregnancy is associated with a unique clinical behavior. It is usually diagnosed in the second or third trimester and in advanced stage. Furthermore, the histological subtype is highly aggressive, and reproductive organ involvement is common. The reduced rate of NHL among females may be explained by direct effects of estrogens on lymphoma cell proliferation or by its effect on anti-tumor immune response. Gender has an important role in responsiveness to standard B cell NHL treatment. Among older adults, women benefited more from the addition of the anti-CD20 antibody rituximab to standard chemotherapy regimens. This phenomenon can be explained by the difference in clearance rate of rituximab that was found to be significantly lower among older females than older males. In mantle cell lymphoma, women receiving lenalidomide have higher rates of response. An understanding of the mechanisms responsible for gender-associated NHL differences will ultimately improve the clinical approach, allowing for a more accurate assessment of prognosis and patient-tailored treatment.

## INTRODUCTION

Lymphoma is a type of cancer that originates from cells of the immune system (i.e. lymphocytes) at different stages of differentiation. Both T cells and B cells can serve as precursors of lymphomas. Unlike leukemia, lymphomas exist mainly in lymph nodes, although the leukemic phase (i.e. lymphoma cells in peripheral blood) is not rare. Today, non-Hodgkin lymphoma (NHL) is one of the most common cancer types worldwide. The etiology of this heterogeneous group of lymphoid malignancies is not well understood, and in most cases remains unknown. The possibility of a multifactorial etiology is appealing, mainly due to the known heterogeneity within this group of malignancies. Correlations between various conditions and lymphoma diagnosis have been found. For example, immunodeficiency states are considered as a predisposition for developing NHL. Autoimmune diseases such as Sjogren syndrome and rheumatoid arthritis have a strong relation to the development of B cell lymphomas.^1^ In addition, the number of NHL subtypes is associated with infectious agents such as EBV, HTLV-I, HIV, HCV, *Helicobacter pylori*, and human herpes virus 8.

The treatment for NHL is based on combination chemotherapy regimens. The addition of rituximab, a monoclonal antibody to CD20, has resulted in prolonged overall survival and better outcomes in terms of complete remission in some lymphoid malignancies. The diversity in response to this treatment among men and women highlights the potential of developing treatment protocols based on gender.^2^

Autologous and allogeneic hematopoietic stem cell transplantation can yield high response rates as front-line or salvage therapies in some lymphomas.

In this review we summarize and discuss the epidemiology, clinical behavior, and response to therapy, focusing on the effect of gender as well as the potential of gender-based therapy for patients with NHL.

## NON-HODGKIN LYMPHOMA INCIDENCE IN RELATION TO GENDER

The incidence rate of NHL has been on the rise since the 1940s, unlike that of Hodgkin lymphoma which has remained stable in the last 20 years.^3^ In the United States, the adjusted incidence is estimated to have increased by 50% from 1970 to 1990.^4^ However, it seems that NHL incidence during the last decade has reached a plateau. For instance, the rates in the United Kingdom between the years 2000 and 2004 were 11.03 per 100,000 for men and 7.87 for women, without a great change in the past decade. Data from an Australian registry revealed that the incidence of NHL increased by 2.5%/year between 1982 and 1996, whereas it remained stable, with an increase of 0.4%/year, from 1997 to 2006.^5^ There has been a decrease in mortality rates in both Europe and the United States (–5.8% and −15.6%, respectively).^6^ In general, women have a 30% lower overall incidence of NHL.^7^

Morton and colleagues analyzed NHL subtype incidence data from the United States National Cancer Institute’s Surveillance, Epidemiology, and End Results (SEER) Program, reported between 1992 and 2001. They found that the incidence rates of Burkitt lymphoma and of diffuse large B cell lymphoma (DLBCL), Sezary syndrome, and many of the T cell lymphomas exhibit a high male/female (M/F) ratio as opposed to other subtypes such as follicular lymphoma.^4^ This finding was further strengthened by a survey conducted in the UK during 2004–2008 that found a M/F ratio of up to 4 in certain subtypes of NHL.^8^

Among adolescents, the statistics reflect the same male predominance. Research conducted in Germany, Austria, and Switzerland between the years 1986 and 2002 reflects the same male predominance with a M/F ratio of 2.7:1 for NHL incidence overall. Burkitt lymphoma showed an even stronger predominance of 4.5:1. Uncommonly, T cell lymphoma demonstrated a higher predominance than B cell lymphoma. T cell lymphoma presented with a ratio of 2.5:1 and DLBCL with a ratio of 1.7:1.^9^

Using the SEER database obtained in Detroit between the years 1973 and 1995, Varterasian and colleagues observed that the adjusted incidence of NHL rose mainly among white and black males. An interesting observation was that the incidence rates were higher in white females in comparison to black males. However, this changed in the late 1980s. The incidence rate for white females rose from 10:100,000 in 1979 to 14:100,000 in 1995, whereas the incidence rate for black males increased from 10:100,000 to 17:100,000. The SEER database lacks further data on other conditions such as immunodeficiency due to HIV that may contribute to this change. Although immunodeficiency can account for some rise in the incidence of NHL (the authors estimate it as a 20% increase), it seems unlikely to be the only factor accounting for this major change.^10^

In addition to female gender itself, gravidity has also been reported to serve a protective role in NHL. Using data from the California Teachers Study (121,004 women included in the analysis), Prescott and colleagues found a relationship between pregnancy and risk reduction for B cell NHL (RR 0.84, 95% CI 0.68–1.04, gravid women to nulligravid women). The reduced risk was relatively constant with increasing number of full-term pregnancies (RR 0.94, 95% CI 0.68–1.31 for one pregnancy, and RR 0.78, 95% CI 0.59–1.02 for more than four pregnancies, *P* for trend 0.001).^3^

## GENDER-RELATED DIFFERENCES IN EXTRA-NODAL INVOLVEMENT

Non-Hodgkin lymphomas (NHL) usually involve the lymphatic system but can also be found in extra-nodal sites. Involvement of extra-nodal sites is a risk factor for poor prognosis according to the most common prognostic tool, the International Prognostic Index (IPI). Using the SEER database, Castillo at el. described extra-nodal involvement in DLBCL (the most common type of B cell NHL). The cohort included 25,992 patients, of whom extra-nodal involvement was diagnosed in 8,204. Extra-nodal disease was found in 31.6% of the patients at the time of diagnosis. These patients were older (62% over the age of 60) and at an earlier stage of disease. The organs most commonly involved were the gastrointestinal tract (34%), head and neck (14%), and skin and soft tissue (11%). There were no specific data regarding central nervous system involvement. Female predominance was found in only 3 of the 12 organs evaluated—breast (96%, *P<*0.001), thyroid (70%, *P*<0.001), and the respiratory system (52%, *P*=0.02). Of these three organs, the involvement of the respiratory system was found to be a poor prognostic factor in terms of overall survival (HR 1.59, *P*<0.001), whereas thyroid and breast involvement were associated with good prognosis (HR 0.79, *P=*0.05, and HR 0.79, *P*=0.08, respectively). No clear predominance was found in the group with head and neck involvement (52%, *P=*0.48).^11^

While breast involvement was found to be a good prognostic factor in lymphoma with extra-nodal localization, the opposite seems to be true for primary breast lymphoma. The term “primary breast lymphoma” (PBL) is used to define a malignant lymphoma primarily occurring in the breast in the absence of previously detected lymphoma localizations. The disease occurs almost exclusively in women. Primary breast lymphoma represents less than 1% of NHL and only about 2% of extra-nodal NHL. Validire et al. analyzed data obtained from 38 women with PBL and histology of DLBCL between 1986 and 2004. Of these patients, 58% were diagnosed with stage IV disease. Significantly lower disease-free and overall survival rates were observed in PBL patients relative to a historical cohort of patients with aggressive nodal lymphoma. A proposed reason for this low rate of response is the fact that only four of the patients in this study received R-CHOP (immuno-chemotherapy protocol; rituximab, cyclophosphamide, doxorubicin, vincristine, prednisone). This might account for such contradicting results.^12^

Similar to PBL in women, “primary testicular lymphoma” (PTL) is an equally rare disease in males, representing only 1%–2% of NHL cases. Unlike PBL, PTL is usually diagnosed early, and up to 90% of patients present with stage I–II disease. When the disease reaches stage III, it is very hard to be distinguished from DLBCL with testicular involvement. Primary testicular lymphoma itself has a propensity for multiple extra-nodal sites, mainly with the involvement of the central nervous system (parenchymal but not leptomeningeal localization), and the disease is characterized by a unique pattern of relapse—most commonly in the central nervous system and the contralateral testis. The administration of R-CHOP, radiation of the contralateral testis, and intrathecal prophylaxis with methotrexate have improved the 5-year overall survival from 56.3% (reported between 1977 and 1999) to 86.6% (reported after 2000).^13^

## NON-HODGKIN LYMPHOMA AND PREGNANCY

The incidence of NHL during pregnancy is particularly low, estimated to be 1 in 6,000 pregnancies. Interestingly, the diagnosis of NHL during pregnancy is associated with a unique clinical behavior. A systematic review including 121 cases of NHL diagnosed during pregnancy was recently published. In this series NHL was usually diagnosed in the second and third trimesters (43% and 42%, respectively). The histological grade of NHL was aggressive (DLBCL and T cell lymphoma) in 48% and highly aggressive (Burkitt lymphoma) in 47% of the cases. This finding shows that a much higher proportion of aggressive lymphoma is prevalent among pregnant women compared to non-pregnant women of reproductive age. Burkitt lymphoma emerged as a common subtype even after excluding endemic Burkitt lymphoma. Stage IV disease was found in 76% of patients, and reproductive organ involvement, including breast, ovary, uterus, and placenta, was reported in 49% of patients. Short-term maternal outcomes were inferior to those reported for young non-pregnant women, at least in the prerituximab era.^14^ A retrospective cohort of 11 academic centers in the United States strengthened these findings. The cohort included 50 patients with NHL of whom 56% were diagnosed with DLBCL, 20% with T cell lymphoma, and 10% with follicular lymphoma. In concordance with the previous study, the rate of advanced disease at diagnosis was high—52% among the NHL patients.^15^

## MECHANISMS UNDERLYING GENDER EFFECTS ON NHL DEVELOPMENT

The influence of sex hormones on lymphoid malignancies has been the subject of clinical and *in vitro* research. Epidemiological studies highlighting the association between sex hormones and NHL have provided some clues. Lee and colleagues reported that an increasing number of pregnancies and live births is associated with a decreasing trend in the risk of DLBCL. They also reported a decreased risk of all NHL subtypes with the use of oral contraceptives (OR 0.68, 95% CI 0.49–0.94). The results were similar for DLBCL and follicular lymphoma (OR 0.75 and 0.79, respectively). Interestingly, other uses of hormones such as hormonal replacement therapy at the age of menopause did not yield the same decrease in overall risk (OR 0.96, 95% CI 0.72–1.3).^7^ The relationship between sex hormones and NHL was further demonstrated by the California Teachers prospective cohort study. Women using oral contraceptives had a lower overall risk of NHL (RR 0.86, 95% CI 0.69–1.06), whereas in women that had undergone bilateral salpingo-oophorectomy and hysterectomy this risk was higher (RR 1.37, 95% CI 1.04–1.8). In this study, hormone replacement therapy was found to have no benefit, and it increased the incidence of follicular lymphoma.^16^ In contrast to the above-mentioned studies, a pooled analysis of InterLymph case–control studies did not find a relationship between NHL and gravidity (pooled OR 0.97, 95% CI 0.8–1.17). However, an increasing number of pregnancies was associated with a lower risk for follicular lymphoma (pooled risk estimated for trend 0.88, 95% CI 0.81–0.96), whereas oral contraceptive use was associated with a higher risk for this disease (pooled OR 1.3, 95% CI 1.04–1.63). The authors speculated that this result might be related to high levels of hormones used in the past in oral contraceptives.^17^ The possibility that other pregnancy-induced factors have an impact on NHL incidence needs to be investigated further.

The molecular mechanisms through which sex hormones influence the development of NHL are not yet fully understood. One possible mechanism may be related to the effect of estrogen on the host immune response.

Rachon et al. showed that 17β-estradiol decreases spontaneous production of interleukin 6 (IL6) by mononuclear cells, resulting in lower serum IL6 levels.^18^ High levels of IL6 in the serum are associated with reduced complete response and diminished overall survival among patients with DLBCL.^19^ Thus, it could be suggested that estrogen provides a protective effect by lowering IL6 levels. Immunological effects not directly related to sex hormones were also explored. T helper cells are vital to human immune response. T helper 1 cells (TH1) secrete cytokines that drive cellular immunity to fight intracellular pathogens and cancerous cells, while T helper 2 cells (TH2) control humoral immunity through up-regulation of antibody production. Imbalanced regulation and expression of TH1 and TH2 cytokines play an important role in the development of NHL. The relationship between polymorphisms in the TH1/TH2 pathway genes, the use of hormonal replacement therapy, and NHL risk was assessed in a case–control study of 597 female patients with NHL and matching controls. It was found that polymorphisms in IL13 (a result of the TH2 pathway) modify the association between hormone replacement therapy and the risk for B cell lymphoma, particularly for follicular lymphoma (reduced risk for IL13 GG, CG, genotypes OR 0.4, 95% CI 0.2–0.9, and increased risk for IL13 AG/AA, CT/TT genotypes OR 2.6, 95% CI 1.2–5.5). The authors suggested that the increased risk was attributed to up-regulation of IL6 by IL13.^20^

Another proposed mechanism is via the direct effect of estrogen on all types of lymphocytes that express the estrogen receptor.^21^ Yakimchuk et al. provided evidence for the anti-proliferative effect of estrogen on lymphoid cells through estrogen receptor β (ERβ) signaling. Exposure of T cell lymphoma and Burkitt lymphoma cells to selective ERβ agonists *in vitro* inhibited their proliferation. In addition, tumor growth was greater in male mice implanted with lymphoma cells compared to female mice. When female mice were ovariectomized the results became almost identical for both genders.^22^ The exact mechanism by which estrogen inhibits lymphoma cell proliferation is still elusive.

## GENDER AND RESPONSE TO THERAPY

The standard therapy for B cell NHL includes multiple courses of combined chemo-immunotherapy. In the last two decades, rituximab, an anti-CD20 chimeral antibody, has become the backbone of this treatment. In T cell lymphoma, multi-agent chemotherapy is the first-line treatment for most entities, while immunotherapy is still investigational and is being evaluated in clinical trials. Gender has an important role in responsiveness to standard treatment, as has been seen mainly in drug protocols including rituximab for B cell NHL.^2^,^23^

The first randomized controlled study assessing R-CHOP (immuno-chemotherapy protocol) versus CHOP (chemotherapy protocol) in patients with DLBCL—the LNH98.5 study conducted in the late 1990s—exhibited the superiority of the R-CHOP regimen. Progression-free survival was 1.2 years in patients receiving CHOP only and 4.8 years in patients receiving R-CHOP (*P*<0.0001). Rituximab was found to reduce the number of refractory patients and the number of patients who relapsed.^24^,^25^ The 2-year survival rate before the rituximab era was estimated to be 64.7% and went up to 85.6% (*P=*0.004) after the introduction of rituximab. In terms of complete response, females had gained a significant advantage due to this combination therapy. The complete response went from 68% to 83.7% for females, but only from 63.9% to 76.6% for males (*P=*0.001).^2^

Sarkozy and colleagues demonstrated that women show a better response to R-CHOP therapy. The authors used data of the LNH-03-2b, LNH-03-6b, and LNH-98-5 studies (all of which are phase III studies of DLBCL treatment incorporating rituximab). The data relate to 985 patients included in these three trials who received R-CHOP. Univariate analysis showed that females have a significantly better progression-free survival (PFS) and a trend for a better overall survival (OS) (HR 1.237, *P=*0.0262, 95% CI 1.025–1.493, and HR 1.218, *P=*0.0681, 95% CI 0.985–1.505, respectively). These differences were most significant in the elderly population (>60 years). Males had a median PFS of 55 months and females 90.6, and an OS of 90.6 and not reached, respectively.^26^

Pfreundschuh and colleagues further investigated this variation using the data obtained in the RICOVER 60, NHL-B2, MInT, and the Mega CHOEP trials. They found that the addition of rituximab to therapy resulted in no difference in response when comparing young males to young females. In contrast, among older adults, women benefited more from the addition of rituximab. This phenomenon can be explained by the difference in clearance rate of rituximab, which was found to be significantly lower among older females than older males (8.47 mL/hour and 10.59 mL/hour, respectively, *P*=0.004).^27^,^28^

The phase II NHL9 study aimed to provide pharmacokinetic data on rituximab during the induction and maintenance phases of treatment. Median levels of rituximab in female patients were higher at almost all time points during induction and maintenance. The researchers found an association between rituximab C_trough_ levels and response to treatment. These results can account for better outcomes among females treated with R-CHOP compared to males.^29^

A few ways to address the pharmacokinetic difference observed between males and females have been proposed, all of which are still under investigation. The SEXIE-R-CHOP-14 trial, using higher doses of rituximab (500 mg/m^2^ as opposed to 375 mg/m^2^), found no significant difference in outcomes between males and females.^30^

Other studies have addressed the subject of the duration of treatment with rituximab. Although no benefit was found for maintenance therapy with rituximab, Murawski et al. showed that among the older population (over 60 years) a longer course of treatment (in which the last dose of rituximab was given at day 239) showed better outcomes than a shorter course (in which the last dose was given at day 99). The study population included 189 patients as part of the SMARTE-R study, and 306 patients that previously participated in the RICOVER-60 study served as controls. Male patients with poor prognosis (*n*=51) were reported to have a 3-year PFS of 71% and 53% (*P=*0.051) and 3-year OS of 80% and 60% (*P=*0.027), respectively. Unlike males, female patients with poor prognosis (*n*=48) had only a minor benefit associated with this prolonged course of treatment, with a 3-year PFS of 71% and 67% (*P=*0.489) and a 3-year OS of 80% and 76% (*P=*0.528), respectively.^31^ Other methods to increase the duration of rituximab activity are under investigation. For example, in the SABRINA trial rituximab is administered subcutaneously and parenterally to create higher and more prolonged serum levels.^32^

Interestingly, gender-associated response to therapy is not limited to rituximab only. Eve and colleagues reported a better outcome in female NHL patients receiving lenalidomide, a drug with anti-proliferative, anti-angiogenic, and immunomodulatory properties. Their phase II study assessed the safety and activity of lenalidomide in 26 patients with relapsed/refractory mantle cell lymphoma, a subgroup of B cell NHL. This study demonstrated that women receiving lenalidomide show better outcomes in terms of complete and partial response (5/7 female patients achieved complete or partial response compared to 3 of 19 male patients, *P=*0.02). Lenalidomide is known to increase the levels of T and NK (natural killer) cells as well as T regulatory cells, probably affecting the immune response against malignant cells. Due to the small study size, the researchers could not address the issue of the relationship between the mechanisms known so far and gender preference.^33^

## CONCLUSION

The current review summarizes differences between male and female NHL patients with respect to disease incidence, clinical characteristics, and response to therapy in the rituximab era. The current knowledge proposed to explain these differences is also discussed. However, there are still many unanswered questions. Understanding of the mechanisms responsible for these differences will ultimately improve the clinical approach, allowing for a more accurate assessment of prognosis and patient-tailored treatment.

## Abbreviations:

DLBCL: diffuse large B cell lymphoma;
IL: interleukin;
IPI: International Prognostic Index;
NHL: non-Hodgkin lymphomas;
NK: natural killer cells;
PBL: primary breast lymphoma;
PFS: progression-free survival;
R-CHOP: rituximab, cyclophosphamide, doxorubicin, vincristine, prednisone;
SEER: United States National Cancer Institute’s Surveillance, Epidemiology, and End Results Program;
TH: T helper cells.
